# Endoscopic ablation versus nephroureterectomy in localized low-grade upper tract urothelial carcinoma: a comparison in terms of cancer-specific and other-cause mortality

**DOI:** 10.1007/s00345-025-05626-0

**Published:** 2025-04-22

**Authors:** Carolin Siech, Letizia Maria Ippolita Jannello, Mario de Angelis, Francesco Di Bello, Natali Rodriquez Peñaranda, Jordan A. Goyal, Zhe Tian, Fred Saad, Shahrokh F. Shariat, Salvatore Micali, Nicola Longo, Ottavio de Cobelli, Alberto Briganti, Benedikt Hoeh, Philipp Mandel, Luis A. Kluth, Felix K. H. Chun, Pierre I. Karakiewicz

**Affiliations:** 1 Cancer Prognostics and Health Outcomes Unit, Division of Urology, University of Montréal Health Center, Montreal, QC Canada; 2 Department of Urology, University Hospital, Goethe University Frankfurt, Theodor-Stern-Kai 7, 60590 Frankfurt am Main, Germany; 3 Department of Urology, IEO European Institute of Oncology, IRCCS, Milan, Italy; 4 Università degli Studi di Milano, Milan, Italy; 5 Unit of Urology, Division of Experimental Oncology, URI, IRCCS Ospedale San Raffaele, Milan, Italy; 6 Vita-Salute San Raffaele University, Milan, Italy; 7 Department of Neuroscience, Science of Reproduction and Odontostomatology, University of Naples Federico II, Naples, Italy; 8 Department of Urology, AOU di Modena, University of Modena and Reggio Emilia, Modena, Italy; 9 Department of Urology, Comprehensive Cancer Center, Medical University of Vienna, Vienna, Austria; 10 Department of Urology, Weill Cornell Medical College, New York, NY USA; 11 Department of Urology, University of Texas Southwestern Medical Center, Dallas, TX USA; 12 Hourani Center for Applied Scientific Research, Al-Ahliyya Amman University, Amman, Jordan; 13 Department of Oncology and Haemato-Oncology, Università degli Studi di Milano, Milan, Italy

**Keywords:** Cancer-specific survival, Other-cause mortality, Kidney-sparing management, UTUC, SEER

## Abstract

**Purpose:**

Guidelines recommend endoscopic ablation in select upper urinary tract urothelial carcinoma (UTUC) patients. To test for differences in cancer-specific mortality (CSM) and other-cause mortality (OCM) in localized non-invasive low-grade UTUC with tumor size < 2 cm treated with endoscopic ablation vs. radical nephroureterectomy.

**Methods:**

Within Surveillance, Epidemiology, and End Results database (2000–2020), we identified UTUC patients treated with either endoscopic ablation or radical nephroureterectomy. After propensity score matching (ratio 1:1), cumulative incidence plots, and competing risks regression models addressed CSM and OCM.

**Results:**

Of 249 included UTUC patients, 66 (27%) were treated with endoscopic ablation vs. 183 (73%) with radical nephroureterectomy. Over the study period, endoscopic ablation use increased from 10 to 45% (p = 0.01). After 1:1 propensity score matching, 66 of 66 (100%) endoscopic ablation and 66 of 183 (36%) radical nephroureterectomy patients were included. Ten-year CSM rates were 15.7% after endoscopic ablation vs. 13.9% after radical nephroureterectomy (p = 0.9). Ten-year OCM rates were 46.3% after endoscopic ablation vs. 57.9% after radical nephroureterectomy (p = 0.5). In multivariable competing risks regression models, CSM (hazard ratio 1.10; p = 0.9) and OCM (hazard ratio 0.83; p = 0.5) did not differ according to use of endoscopic ablation vs. radical nephroureterectomy.

**Conclusion:**

Endoscopic ablation of localized non-invasive low-grade UTUC with tumor size < 2 cm results in absence of cancer-control outcome differences relative to radical nephroureterectomy. This observation validates the current guideline recommendations.

**Supplementary Information:**

The online version contains supplementary material available at 10.1007/s00345-025-05626-0.

## Introduction

Upper urinary tract urothelial carcinoma (UTUC) represents a rare urological malignancy, accounting for only 5–10% of urothelial carcinoma cases [1]. Its age-standardized incidence rates are as low as 1.3 cases per 100,000 people per year [2]. UTUC is twice as common in males than in females [3] and predominantly originates in the renal pelvis, compared to the ureteral location [4, 5]. Even in non-metastatic UTUC patients, the estimated 5-year cancer-specific survival (CSS) rate is only 27%, making UTUC to a highly aggressive disease [2].

Radical nephroureterectomy represents the standard of care for the majority of non-metastatic UTUC patients [6, 7]. However, radical surgery is associated with considerable morbidity, including impairment in renal function and risk of end-stage renal disease [8–11]. Therefore, European and North American guidelines recommend kidney-sparing surgery, such as endoscopic ablation, as first-line treatment in select patients with non-metastatic low-risk disease when technically feasible [6, 7]. Selection criteria for low-risk UTUC patients include unifocal disease, tumor size < 2 cm, negative for high-grade cytology, low-grade ureterorenoscopy biopsy, and non-invasive tumor stage on computer tomographic scan [6, 7]. Although these selection criteria have already been introduced by the European Association of Urology (EAU) in 2017 [6], no large-scale population-based study validated that endoscopic ablation does not undermine cancer-specific mortality (CSM)-free rates when applicable. Previous studies either did not meet the guideline-defined inclusion criteria for low-risk UTUC patients [12–14] or focused on different study endpoints, such as overall mortality (OM) instead of CSM [15].

We addressed this knowledge gap and hypothesized that endoscopic ablation use has increased over time in select UTUC patients. Additionally, we also postulated that CSM-free survival is not inferior in patients treated with endoscopic ablation relative to their radical nephroureterectomy counterparts. Finally, we examined the importance of OCM to corroborate the central role of CSM as an endpoint in survival analyses of this select patient group in whom OCM may largely outweigh and confound survival analyses that rely on OM. To test these hypotheses, we relied on a population-based cohort of select UTUC patients treated with endoscopic ablation or radical nephroureterectomy in the United States between 2000 and 2020.

## Materials and methods

### Data source and study population

SEER database (2000–2020) provides cancer statistics covering approximately 47.9% of the United States population [16]. Within the SEER database, we identified newly diagnosed histologically confirmed UTUC (International Classification of Diseases [ICD-10] site codes C65 and C66) patients. Inclusion criteria consisted of age ≥ 18 years, known vital status, known cause of death, and localized stage. Autopsy- or death certificate-only cases were excluded. Based on guideline-recommended risk stratification in non-metastatic UTUC [6], only patients with non-invasive low-grade UTUC with tumor size < 2 cm treated with either endoscopic ablation, defined as electrocautery, cryosurgery, laser ablation or excision, or local tumor excision not otherwise specified (Surgery site codes 12–14, and 20, 22–25, and 27) or radical nephroureterectomy (Surgery site codes 40 and 50) were selected for the current study cohort. Other surgical procedures, such as partial nephrectomy or segmental ureterectomy were excluded (Supplementary Fig. 1). Due to the anonymously coded design of the SEER database, study-specific Institutional Review Board ethics approval was not required. The study has been conducted in accordance with the principles set in the Helsinki Declaration.

### Study endpoints

The primary study endpoint represented cancer-specific mortality (CSM) defined as death from UTUC. The secondary endpoint consisted of other-cause mortality (OCM) defined as death due to any cause except for mortality from UTUC in accordance with the SEER mortality code.

### Statistical analyses

First, baseline characteristics were tabulated. Descriptive statistics included medians and interquartile ranges (IQR) for continuously coded variables and frequencies and proportions for categorical variables. Wilcoxon rank sum test examined the statistical significance of medians’ differences for continuously coded variables. Pearson’s Chi-square test assessed the statistical significance in proportions’ differences for categorical variables. Second, Estimated Percentage Increase (EPI) per three years was calculated for endoscopic ablation use with the least squares linear regression. Third, multivariable logistic regression models were used to test the association between patient and tumor characteristics and treatment with endoscopic ablation. Fourth, propensity score matching (ratio 1:1) according to the nearest neighbor was applied for age at diagnosis and tumor size between endoscopic ablation and radical nephroureterectomy patients to maximally reduce the effect of bias and confounding [17]. Fifth, cumulative incidence plots displayed CSM and OCM of UTUC patients treated with endoscopic ablation vs. radical nephroureterectomy. Subsequently, multivariable competing risks regression models were fitted to test for CSM and OCM differences according to endoscopic ablation vs. radical nephroureterectomy. Adjustment variables consisted of age at diagnosis (years, continuously coded), sex (female vs. male), tumor size (cm, continuously coded), and tumor location (ureter vs. renal pelvis). Statistical tests were two sided with a level of significance set at p < 0.05. R software environment was used for statistical computing and graphics (R version 4.3.2; R Foundation for Statistical Computing, Vienna, Austria) [18].

## Results

### Descriptive characteristics and temporal trends

**Fig. 1:**
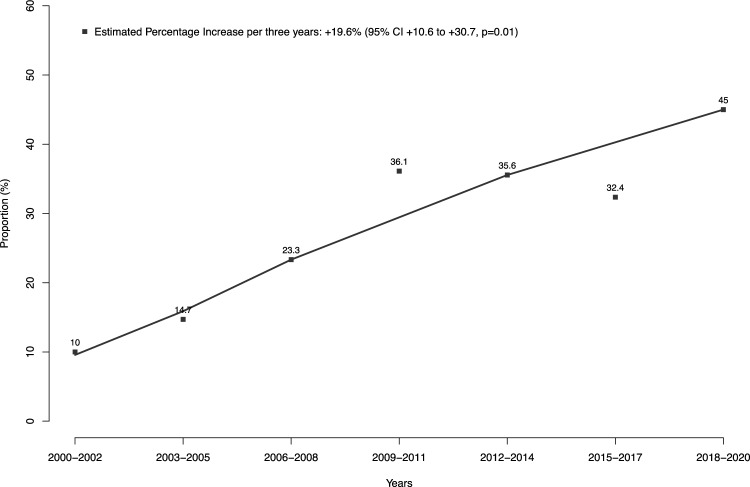
Estimated Percentage Increase (EPI) per three years of endoscopic ablation use in surgically-treated localized non-invasive low-grade upper urinary tract urothelial carcinoma (UTUC) patients with tumor size < 2 cm between 2000 and 2020. *CI* confidence interval

Of 249 localized non-invasive low-grade UTUC patients with tumor size < 2 cm, 66 (27%) were treated with endoscopic ablation vs. 183 (73%) were treated with radical nephroureterectomy (Supplementary Fig. 1). Among the 66 patients treated with endoscopic ablation, 13 (19.7%) underwent electrocautery, 1 (1.5%) underwent cryosurgery, 35 (53.0%) underwent laser ablation, and 7 (10.6%) underwent laser excision. The mode of endoscopic ablation was not further specified for 10 (15.2%) patients. Over the study period, endoscopic ablation use increased from 10 to 45% among surgically-treated patients (EPI per three years + 19.6%, 95% confidence interval [CI] + 10.6 to 30.7%; p = 0.01; Fig. 1). Relative to radical nephroureterectomy patients, endoscopic ablation patients were older (median age 77 vs. 73 years, IQR 70–83 vs. 64–77 years; p < 0.001) and harbored smaller tumors (median tumor size 1.0 vs. 1.5 cm, IQR 0.9–1.5 vs. 1.0–1.5 cm; p < 0.001). Conversely, no statically significant differences were observed for sex and tumor location (Table 1). Likewise, in multivariable logistic regression models, both older age (OR 1.07, 95% CI 1.04–1.11; p < 0.001) and smaller tumor size (OR 0.30, 95% CI 0.15–0.60; p < 0.001) independently predicted treatment with endoscopic ablation relative to radical nephroureterectomy. No statistically significant association with administered treatment was observed for sex (female vs. male sex: OR 0.93, 95% 0.50–1.73; p = 0.8) and tumor location (ureter vs. renal pelvis: OR 1.10, 95% 0.60–2.03; p = 0.8).

**Table 1:** Descriptive characteristics of 249 localized non-invasive low-grade upper urinary tract urothelial carcinoma (UTUC) patients with tumor size < 2 cm stratified according to treatment (endoscopic ablation vs. radical nephroureterectomy) prior to and after propensity score matching (ratio 1:1)

	Prior propensity score matching				After propensity score matching^c^		
Characteristic	Overall, n = 249^a^	Endoscopic ablation, n = 66 (27%)^a^	Radical nephro-ureterectomy, n = 183 (73%)^a^	p value^b^	Endoscopic ablation, n = 66 (50%)^a^	Radical nephro-ureterectomy, n = 66 (50%)^a^	p value^b^
Age at diagnosis (in years)	73 (65, 79)	77 (70, 83)	73 (64, 77)	** < 0.001**	77 (70, 83)	75 (73, 81)	0.6
Male sex	154 (62%)	41 (62%)	113 (62%)	1.0	41 (62%)	42 (64%)	0.9
Tumor size (in cm)	1.4 (1.0, 1.5)	1.0 (0.9, 1.5)	1.5 (1.0, 1.5)	** < 0.001**	1.0 (0.9, 1.5)	1.2 (0.7, 1.5)	0.8
Tumor location				0.6			0.9
Renal pelvis	151 (61%)	38 (58%)	113 (62%)		38 (58%)	39 (59%)	
Ureter	98 (39%)	28 (42%)	70 (38%)		28 (42%)	27 (41%)	

^a^Median (interquartile range); n (%)

^b^Wilcoxon rank sum test; Pearson's Chi-square test; Fisher's exact test

^c^Propensity score matching (ratio 1:1) for age at diagnosis and tumor size

Relying on 1:1 propensity score matching for age at diagnosis and tumor size, of 249 UTUC patients, 66 of 66 (100%) patients treated with endoscopic ablation and 66 of 183 (36%) patients treated with radical nephroureterectomy were included in further analyses. After propensity score matching, no statistically significant residual differences remained for all above variables (Table 1).

### Cancer-specific mortality rates after propensity score matching

**Fig. 2:**
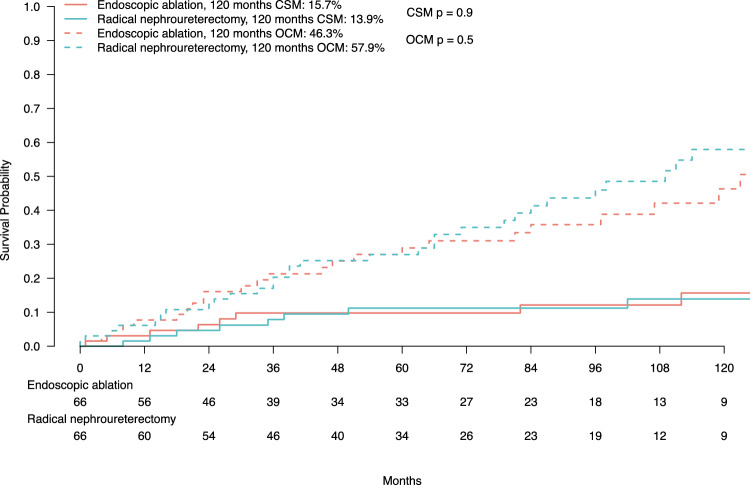
Cumulative incidence plot addressing cancer-specific mortality (CSM) and other-cause mortality (OCM) of localized non-invasive low-grade upper urinary tract urothelial carcinoma (UTUC) patients with tumor size < 2 cm according to treatment (endoscopic ablation vs. radical nephroureterectomy) after propensity score matching (ratio 1:1). *CSM* cancer-specific mortality, *OCM* other-cause mortality

Of all 66 endoscopic ablation patients, 8 (12.1%) died of UTUC. This was reflected in a ten-year CSM rate of 15.7% (Fig. 2). Similarly, of all 66 radical nephroureterectomy patients, 8 (12.1%) died of UTUC. This was reflected in a ten-year CSM rate of 13.9% (CSM Δ1.8%). These rates resulted in a univariable hazard ratio (HR) for CSM of 1.08 (95% CI 0.41–2.83; p = 0.9; Table 2). After multivariable adjustment for age at diagnosis, sex, tumor size, and tumor location and additional accounting for OCM, the multivariable HR for CSM remained at 1.10 (95% CI 0.42–2.87; p = 0.9; Table 2).

**Table 2:** Univariable and multivariable competing risks regression (CRR) models addressing cancer-specific mortality (CSM) and other-cause mortality (OCM) in localized non-invasive low-grade urinary tract urothelial carcinoma (UTUC) patients with tumor size < 2 cm, according to treatment (endoscopic ablation vs. radical nephroureterectomy) after propensity score matching (ratio 1:1)

	Univariable		Multivariable^a^	
	HR (95% CI)	p value	HR (95% CI)	p value
CSM				
Endoscopic ablation vs. Radical nephroureterectomy	1.08 (0.41, 2.83)	0.9	1.10 (0.42, 2.87)	0.9
OCM				
Endoscopic ablation vs. Radical nephroureterectomy	0.82 (0.50, 1.33)	0.4	0.83 (0.51, 1.36)	0.5

*CI* confidence interval, *CSM* cancer-specific mortality, *HR* hazard ratio, *OCM* other-cause mortality

^a^Adjusted for age at diagnosis, sex, tumor size, and tumor location

### Other-cause mortality rates after propensity score matching

Of all 66 endoscopic ablation patients, 27 (40.9%) died of other causes. This was reflected in a ten-year OCM rate of 46.3% (Fig. 2). Conversely, of all 66 radical nephroureterectomy patients, 35 (53%) died of other causes. This was reflected in a ten-year OCM rate of 57.9% (OCM Δ−11.6%). These rates resulted in an univariable HR for OCM of 0.82 (95% CI 0.50–1.33; p = 0.4; Table 2). After multivariable adjustment for age at diagnosis, sex, tumor size, and tumor location and additional accounting for CSM, the multivariable HR for OCM remained at 0.83 (95% CI 0.51–1.36; p = 0.5; Table 2).

## Discussion

Guidelines recommend endoscopic ablation in select UTUC patients, including those with non-invasive tumor stage, low-grade histology, and tumor size < 2 cm [6, 7]. However, no large-scale population-based study validated that endoscopic ablation does not undermine CSM-free rates in select UTUC patients. We addressed this knowledge gap and made several noteworthy observations.

First, UTUC is a rare cancer [1, 6, 7]. Among all stages of UTUC, those considered for endoscopic ablation, namely localized non-invasive low-grade UTUC with tumor size < 2 cm represent even a rarer entity. Specifically, Upfill-Brown et al. relied on the National Cancer Database (NCDB) and identified 851 patients [15]. This highly select patient cohort accounted only for 2.0% of the overall population of 43,036 UTUC patients within the NCDB between 2004 and 2012 [15]. In the current study, we identified 249 localized non-invasive low-grade UTUC patients with tumor size < 2 cm over a 21-year period within the SEER database (2000–2020). In consequence, select UTUC patients should ideally be included in multi-institutional studies or large population-based analyses, as was done in the current study, when cancer-control outcomes represent the outcome of interest in this small subgroup of a rare primary tumor.

Second, we stratified the study cohort of localized non-invasive low-grade UTUC with tumor size < 2 cm according to surgical treatment type: endoscopic ablation vs. radical nephroureterectomy. Of 249 patients that fulfilled the above selection criteria, as many as 66 benefited from endoscopic ablation. The proportion of patients having benefitted from endoscopic ablation between 2000 and 2020 within the current study is only 27%, which is lower than 38% previously reported by Upfill-Brown et al. based on NCDB [15]. However, the rate of endoscopic ablation increased to 45% in the three most recent years of the current study (EPI + 19.6%; p = 0.01). This observation is highly encouraging. Unfortunately, it cannot be directly compared to the analysis of Upfill-Brown et al. since NCDB data addressing the select subgroup of patients with localized non-invasive low-grade UTUC with tumor size < 2 cm were not tabulated according to years of diagnosis [15]. However, tabulations of endoscopic ablation rates over time in a cohort that relied on wider selection criteria than used in the current study also increased over time [15, 19]. These increases might be explained, among other factors, by broader indication for endoscopic ablation in recent UTUC guidelines [15], technical advances in endourology [20], as well as greater availability of endourological experts than in historical years. Additionally, the increasing rates of endoscopic ablation also indicate increasing acceptance over time of guideline recommendations within the urologic community.

Third, we identified important differences in baseline characteristics between endoscopic ablation vs. radical nephroureterectomy patients. Specifically, endoscopic ablation patients were older (median age 77 vs. 73 years) and harbored smaller tumors (median tumor size 1.0 vs. 1.5 cm) than their counterparts treated with radical nephroureterectomy. Based on these differences, it is essential to rely on propensity score matching (endoscopic ablation vs. radical nephroureterectomy patients) to reduce or ideally eliminate uncontrolled confounding or bias originating from patient and tumor characteristics, as was done in the present study. After detailed propensity score matching for age at diagnosis and tumor size that resulted in minimal cohort differences, 66 of 66 (100%) endoscopic ablation and 66 of 183 (36%) radical nephroureterectomy patients remained. Indeed, the use of propensity score matching virtually perfectly eliminated meaningful differences between endoscopic ablation vs. radical nephroureterectomy patients. However, other patient characteristics, such as performance status and comorbidities (e.g. cardiovascular disease or chronic kidney disease), which may influence treatment decision-making due to elevated risk of perioperative complications, could not be considered as covariates in the present study, as the current version of the SEER database does not provide this information [21]. Moreover, an important attrition in the size of the nephroureterectomy cohort was also recorded, without concomitant loss of observations within the endoscopic ablation cohort. Such phenomenon unfortunately invariably accompanies the use of propensity score matching.

Fourth, we addressed CSM as cancer-control endpoint. Specifically, we tested for CSM differences in localized non-invasive low-grade UTUC patients with tumor size < 2 cm treated with endoscopic ablation vs. radical nephroureterectomy. In survival analyses that relied on propensity score matched cohorts of endoscopic ablation and radical nephroureterectomy patients (66:66), ten-year CSM rates were 15.7% after endoscopic ablation vs. 13.9% after radical nephroureterectomy (CSM Δ1.8%; p = 0.9). In competing risks regression models that accounted for OCM and relied on propensity score matching as well as additional multivariable adjustment, these rates translated into a HR of 1.10 (95% CI 0.42–2.87; p = 0.9). Since previous studies either did not meet the guideline-defined inclusion criteria for low-risk UTUC patients [12–14] or, if they did, addressed different study endpoints, such as OM instead of CSM [15], no direct comparison of the current results with previous studies can be made. However, these results among select low-risk UTUC patients convincingly illustrate that CSM rates, which may be expected after endoscopic ablation, are clearly not inferior to those observed after radical nephroureterectomy. Patients and clinicians should consider that endoscopic treatment is associated with additional benefits compared to radical nephroureterectomy, such as shorter hospital stay, fewer Clavien–Dindo high-grade postoperative complications, and less decline in estimated glomerular filtration rate (eGFR) [8]. Furthermore, progression to dialysis-dependent kidney disease is associated with a decrease in quality of life as well as higher health costs [22]. In consequence, endoscopic ablation may be offered and preferred not only at centers of excellence with established track records of kidney-sparing management in low-risk UTUC patients, but also in population-based settings when technically feasible.

Finally, the current study also addressed OCM in localized non-invasive low-grade UTUC patients with tumor size < 2 cm as a secondary endpoint. Absolute ten-year OCM rates of 46.3 vs. 57.9% (ΔOCM 11.6%; p = 0.5) were recorded after endoscopic ablation vs. radical nephroureterectomy. These rates illustrate that the majority of mortality events represented non-cancer-related deaths. Specifically, of all deaths in patients treated with endoscopic ablation 27 of 35 were OCM events. Similarly, of all deaths in patients treated with radical nephroureterectomy 35 of 43 represented OCM events. The phenomenon of higher OCM than CSM rates may be explained in several ways. First, low CSM rates may be attributed to the low-grade nature of UTUC examined in the present study, making it more likely to die from other health conditions (e.g., cardiovascular diseases) before UTUC progresses to a fatal stage. Second, UTUC occurs in elderly patients. Age-related decline and frailty make the UTUC patient cohort susceptible to other causes of death. Third, treatment-related morbidity may be another explanation. However, the observation of lower CSM and higher OCM rates applies to both treatment groups (endoscopic ablation and radical nephroureterectomy). Therefore, it is more likely that the observation of lower rates of CSM and higher rates of OCM may be attributed to the nature of low-risk UTUC, rather than to differences between the two patient cohorts or delivered treatments. However, the SEER database is not detailed enough to allow more definitive assessment of those associations. In consequence, the proposed explanations remain preliminary at its best.

Nevertheless, lack of specific CSM consideration in previous analyses may have resulted in erroneous consideration of the majority of deaths in analyses that attempted to quantify cancer control outcomes. Possibly, in NCDB analyses, where endoscopic ablation was interpreted as a treatment option with worse survival outcomes compared to radical nephroureterectomy, the methodological flow where OM instead of CSM is considered as study endpoint, may have contributed to this potential misinterpretation [15]. This potential misinterpretation of treatment comparisons when OM is used instead of CSM as the study endpoint has already been demonstrated for other treatment comparisons, such as trimodal therapy vs. external beam radiotherapy in non-metastatic urothelial carcinoma of the bladder, as well as radiotherapy vs. partial penectomy in localized penile cancer [23, 24]. In these cases, reliance on OM alone would have led to misleading conclusions regarding treatment efficacy, as differences in mortality were influenced by competing risks rather than cancer-related deaths. Therefore, CSM represents an essential survival endpoint in treatment comparisons in localized non-invasive low-grade UTUC patients with tumor size < 2 cm since CSM only accounts for a small proportion of OM events and the competing and potentially confounding OCM effect accounts for the remaining majority of OM events.

Taken together, the proportion of patients undergoing endoscopic ablation increased from 10 to 45%, reflecting growing confidence in endoscopic management for select UTUC patients. After strict methodology, including propensity score matching, multivariable adjustment, and accounting for OCM, CSM rates after endoscopic ablation vs. radical nephroureterectomy neither showed statistically significant or clinically meaningful differences. These observations among North American UTUC patients provide a large-scale contemporary validation of the current European guideline recommendations to identify low-risk UTUC patients eligible for endoscopic ablation.

Despite its novelty, the present study is not devoid of limitations. First, the current study shares the limitations of all UTUC studies that used an observational study design and a retrospective database, such as SEER [2, 12, 13, 25–31], NCDB [15, 19] or multi-institutional databases [8, 10, 14, 32]. Even after strict and systematic adjustment for confounders and biases relying on propensity score matching, multivariable adjustment and accounting for OCM or CSM in competing risks regression models, a potential for selection biases remained. Second, despite the large scale of the SEER database, the sample size within the SEER database is limited due to the rarity of localized non-invasive low-grade UTUC with tumor size < 2 cm. Therefore, subgroup analyses according to specific endoscopic procedures, such as electrocautery or laser ablation were not possible. Third, the current study focuses on the comparison of endoscopic ablation vs. radical nephroureterectomy. Other kidney-sparing surgical techniques, such as partial nephrectomy or segmental ureterectomy, which may also be considered in select UTUC patients were not included [33]. Fourth, patient clinical characteristics, such as performance status or comorbidities (e.g. cardiovascular disease or chronic kidney disease) are not available in the current version of the SEER database. Therefore, these patient characteristics that may have influenced treatment decision cannot be considered as matching variables or for further adjustments in multivariable models in the present study [31]. However, lack of comorbidities was at least in part addressed with competing risks regression analyses that accounted for OCM since most important comorbidities result in OCM. Fifth, the SEER database only provides a limited amount of detail regarding tumor characteristics and procedural details. Unfortunately, the number and frequency of endoscopic ablations cannot be analyzed. Likewise, drug-specific information regarding postoperative or adjuvant intravesical instillation therapies is not available. Moreover, the current version of the SEER database only provides tumor morphology and stage at initial diagnosis. Therefore, data on non-organ-confined tumors (pT3/pT4), positive lymph nodes (pN +), and positive surgical margins (R1) are unavailable for endoscopic ablation patients who later underwent radical nephroureterectomy. Additionally, detailed information regarding tumor appearance, such as tumor focality, pathological characteristics, such as urinary cytology, as well as clinical characteristics, such as hydronephrosis are unknown. Nevertheless, all available risk stratification variables for non-metastatic UTUC patients, provided by the SEER database, were considered as inclusion criteria for the selection of the current study cohort. Finally, the SEER database does not include earlier cancer-control endpoints than CSM and OCM. In consequence, other study endpoints that could be equally as interesting as CSM, such as intravesical recurrence or metastasis could not be addressed within the present database.

## Conclusions

Endoscopic ablation of localized non-invasive low-grade UTUC with tumor size < 2 cm results in absence of cancer-control outcome differences relative to radical nephroureterectomy. This observation validates the current guideline recommendations.

## Supplementary Information

Below is the link to the electronic supplementary material.

Supplementary file1 (DOCX 177 KB)

## Data Availability

All analyses and their reporting followed the SEER reporting guidelines. The data used in this study (SEER 2004–2020) is available in the SEER Incidence Data 1975–2020 repository, https://seer.cancer.gov/data/. Further information is available from the corresponding author upon request.

## References

[1] Siegel RL, Miller KD, Wagle NS, Jemal A (2023) Cancer statistics, 2023. CA: Cancer J Clin 73:17–48. 10.3322/caac.21763 36633525 10.3322/caac.21763

[2] Collà Ruvolo C, Nocera L, Stolzenbach LF, Wenzel M, Cucchiara V, Tian Z et al (2021) Incidence and survival rates of contemporary patients with invasive upper tract urothelial carcinoma. Eur Urol Oncol 4:792–801. 10.1016/j.euo.2020.11.005 33293235 10.1016/j.euo.2020.11.005

[3] Shariat SF, Favaretto RL, Gupta A, Fritsche H-M, Matsumoto K, Kassouf W et al (2011) Gender differences in radical nephroureterectomy for upper tract urothelial carcinoma. World J Urol 29:481–486. 10.1007/s00345-010-0594-7 20886219 10.1007/s00345-010-0594-7

[4] Collà Ruvolo C, Deuker M, Wenzel M, Nocera L, Würnschimmel C, Califano G et al (2022) Impact of the primary tumor location on secondary sites and overall mortality in patients with metastatic upper tract urothelial carcinoma. Urol Oncol: Semin Orig Investig 40:411.e1-411.e8. 10.1016/j.urolonc.2022.06.009 10.1016/j.urolonc.2022.06.009 35902301

[5] Lwin AA, Hsu C-H, Chipollini J (2020) Urothelial carcinoma of the renal pelvis and ureter: does location make a difference? Clin Genitourin Cancer 18:45-49.e1. 10.1016/j.clgc.2019.10.023 31786118 10.1016/j.clgc.2019.10.023

[6] Rouprêt M, Seisen T, Birtle AJ, Capoun O, Compérat EM, Dominguez-Escrig JL et al (2023) European association of urology guidelines on upper urinary tract urothelial carcinoma: 2023 update. Eur Urol 84:49–64. 10.1016/j.eururo.2023.03.013 36967359 10.1016/j.eururo.2023.03.013

[7] Coleman Jonathan A, Clark Peter E, Bixler Brooke R, Buckley David I, Chang Sam S, Chou R et al (2023) Diagnosis and management of non-metastatic upper tract urothelial carcinoma: AUA/SUO guideline. J Urol 209:1071–1081. 10.1097/JU.0000000000003480 37096584 10.1097/JU.0000000000003480

[8] Chen Y-T, Yu C-C, Yeh H-C, Lee H-Y, Jiang Y-H, Lee Y-K et al (2021) Endoscopic management versus radical nephroureterectomy for localized upper tract urothelial carcinoma in a high endemic region. Sci Rep 11:4040. 10.1038/s41598-021-83495-4 33597574 10.1038/s41598-021-83495-4 PMC7889610

[9] Shen C-Y, Jou Y-C, Kan W-C, Tzai T-S, Tsai Y-S (2022) Outcome of Non-Muscle Invasive Upper Tract Urothelial Carcinoma Receiving Endoscopic Ablation: An Inverse Probability of Treatment Weighting Analysis. J Clin Med. 10.3390/jcm11051307 35268398 10.3390/jcm11051307 PMC8910842

[10] Tsujino T, Komura K, Inamoto T, Maenosono R, Hashimoto T, Adachi T et al (2023) Nephron-sparing ureteroscopic surgery vs. radical nephroureterectomy: comparable survival-outcomes in upper tract urothelial carcinoma. World J Urol 41:3585–3591. 10.1007/s00345-023-04687-3 37924336 10.1007/s00345-023-04687-3

[11] Hoffman A, Yossepowitch O, Erlich Y, Holland R, Lifshitz D (2014) Oncologic results of nephron sparing endoscopic approach for upper tract low grade transitional cell carcinoma in comparison to nephroureterectomy: a case control study. BMC Urol 14:97. 10.1186/1471-2490-14-97 25468319 10.1186/1471-2490-14-97 PMC4265434

[12] Ye Y, Zheng Y, Li J, Miao Q, Lin M, Chen J et al (2023) Endoscopic excision versus radical nephroureterectomy for non-muscle invasive upper tract urothelial carcinoma: a population-based large cohort study. Heliyon. 10.1016/j.heliyon.2023.e22408 38107280 10.1016/j.heliyon.2023.e22408 PMC10724554

[13] Qiu J, Deng R, Yu C, Gong K (2023) The long-term outcome of nephron-sparing surgery versus radical nephroureterectomy for organ-localized upper urinary tract urothelial carcinoma: a population-based study of 1969 patients. J Cancer Res Clin Oncol 149:14869–14878. 10.1007/s00432-023-05264-2 37598342 10.1007/s00432-023-05264-2 PMC11797156

[14] Chen YT, Yeh H-C, Lee H-Y, Hsieh P-F, Chou EC, Tsai Y-C et al (2023) Endoscopic management of upper tract urothelial cancer in a highly endemic area: a Taiwan nationwide collaborative study. Asian J Surg 46:3058–3065. 10.1016/j.asjsur.2022.10.046 37525448 10.1016/j.asjsur.2022.10.046

[15] Upfill-Brown A, Lenis AT, Faiena I, Salmasi AH, Johnson DC, Pooli A et al (2019) Treatment utilization and overall survival in patients receiving radical nephroureterectomy versus endoscopic management for upper tract urothelial carcinoma: evaluation of updated treatment guidelines. World J Urol 37:1157–1164. 10.1007/s00345-018-2506-1 30267197 10.1007/s00345-018-2506-1 PMC6438772

[16] National Cancer Institute. SEER Incidence Data, 1975–2020. National Institutes of Health 2023. https://seer.cancer.gov/data/. Accessed 8 Feb 2024

[17] Austin PC (2011) An introduction to propensity score methods for reducing the effects of confounding in observational studies. Multivar Behav Res 46:399–424. 10.1080/00273171.2011.568786 10.1080/00273171.2011.568786 PMC3144483 21818162

[18] R Core Team. R: A Language and Environment for Statistical Computing. R: A Language and Environment for Statistical Computing 2022. https://www.R-project.org/. Accessed 27 Aug 2023

[19] Browne BM, Stensland KD, Moynihan MJ, Canes D (2018) An analysis of staging and treatment trends for upper tract urothelial carcinoma in the national cancer database. Clin Genitourin Cancer 16:e743–e750. 10.1016/j.clgc.2018.01.015 29506950 10.1016/j.clgc.2018.01.015

[20] Cornu J-N, Rouprêt M, Carpentier X, Geavlete B, de Medina SGD, Cussenot O et al (2010) Oncologic control obtained after exclusive flexible ureteroscopic management of upper urinary tract urothelial cell carcinoma. World J Urol 28:151–156. 10.1007/s00345-009-0494-x 20044752 10.1007/s00345-009-0494-x

[21] Di Bello F, Rodriguez Peñaranda N, Siech C, de Angelis M, Tian Z, Goyal JA et al (2024) Perioperative complications and in-hospital mortality in radical nephroureterectomy patients with heart valve replacement. Ann Surg Oncol. 10.1245/s10434-024-16639-1 39627641 10.1245/s10434-024-16639-1

[22] Suriano F, Brancato T (2014) Nephron-sparing management of upper tract urothelial carcinoma. Rev Urol 16:21–28 24791152 PMC4004281

[23] Jannello LMI, Siech C, de Angelis M, Di Bello F, Rodriguez Peñaranda N, Tian Z et al (2024) Radiotherapy versus partial penectomy for T1 squamous cell carcinoma of the penis. Ann Surg Oncol. 10.1245/s10434-024-15767-y 38980582 10.1245/s10434-024-15767-y

[24] de Angelis M, Siech C, Di Bello F, Rodriguez Peñaranda N, Goyal JA, Tian Z et al (2024) Survival rates in trimodal therapy versus radiotherapy in urothelial carcinoma of urinary bladder. Eur Urol Focus. 10.1016/j.euf.2024.09.013 39366885 10.1016/j.euf.2024.09.013

[25] Simhan J, Smaldone MC, Egleston BL, Canter D, Sterious SN, Corcoran AT et al (2014) Nephron-sparing management vs radical nephroureterectomy for low- or moderate-grade, low-stage upper tract urothelial carcinoma. BJU Int 114:216–220. 10.1111/bju.12341 24053485 10.1111/bju.12341 PMC4486373

[26] Vemana G, Kim EH, Bhayani SB, Vetter JM, Strope SA (2016) Survival comparison between endoscopic and surgical management for patients with upper tract urothelial cancer: a matched propensity score analysis using surveillance, epidemiology and end results-Medicare data. Urology 95:115–120. 10.1016/j.urology.2016.05.033 27233931 10.1016/j.urology.2016.05.033 PMC5115634

[27] Ślusarczyk A, Zapała P, Zapała Ł, Rajwa P, Moschini M, Laukhtina E et al (2024) Oncologic outcomes of patients treated with kidney-sparing surgery or radical nephroureterectomy for upper urinary tract urothelial cancer: a population-based study. Urol Oncol: Semin Orig Investig 42:22.e1-22.e11. 10.1016/j.urolonc.2023.09.019 10.1016/j.urolonc.2023.09.019 37981503

[28] Fero KE, Shan Y, Lec PM, Sharma V, Srinivasan A, Movva G et al (2021) Treatment patterns, outcomes, and costs associated with localized upper tract urothelial carcinoma. JNCI Cancer Spectr. 10.1093/jncics/pkab085 34805743 10.1093/jncics/pkab085 PMC8599752

[29] Di Bello F, Jannello LMI, Siech C, de Angelis M, Rodriguez Peñaranda N, Tian Z et al (2024) Adjuvant systemic therapy improved survival after radical nephroureterectomy for upper tract urothelial carcinoma. Ann Surg Oncol. 10.1245/s10434-024-15814-8 39031261 10.1245/s10434-024-15814-8

[30] Di Bello F, de Angelis M, Siech C, Peñaranda NR, Tian Z, Goyal JA et al (2025) Non-Caucasian race/ethnicity predisposes to unfavorable stage and grade at upper tract urothelial carcinoma diagnosis. J Racial Ethn Health Disparities. 10.1007/s40615-025-02285-0 39789348 10.1007/s40615-025-02285-0 PMC12966194

[31] Di Bello F, Siech C, de Angelis M, Rodriguez Peñaranda N, Jannello LMI, Tian Z et al (2025) Bladder cuff excision at radical nephroureterectomy improved survival in upper tract urothelial carcinoma. Urol Oncol: Semin Orig Investig. 10.1016/j.urolonc.2025.02.014 10.1016/j.urolonc.2025.02.014 40118680

[32] Rouprêt M, Hupertan V, Traxer O, Loison G, Chartier-Kastler E, Conort P et al (2006) Comparison of open nephroureterectomy and ureteroscopic and percutaneous management of upper urinary tract transitional cell carcinoma. Urology 67:1181–1187. 10.1016/j.urology.2005.12.034 16765178 10.1016/j.urology.2005.12.034

[33] Paciotti M, Alkhatib KY, Nguyen D-D, Yim K, Lipsitz SR, Mossanen M et al (2023) Is segmental ureterectomy associated with inferior survival for localized upper-tract urothelial carcinoma of the ureter compared to radical nephroureterectomy? Cancers. 10.3390/cancers15051373 36900166 10.3390/cancers15051373 PMC10000204

