# Supplementary material for: Endoscopic ablation versus nephroureterectomy in localized low-grade upper tract urothelial carcinoma: a comparison in terms of cancer-specific and other-cause mortality
Source: World J Urol. 2025 Apr 22;43(1):241. doi: 10.1007/s00345-025-05626-0 (PMC12014723; doi:10.1007/s00345-025-05626-0)

**Supplementary Figure 1** Consort diagram

Abbreviations: SEER= Surveillance, Epidemiology, and End Results database; UTUC= upper urinary tract urothelial carcinoma.


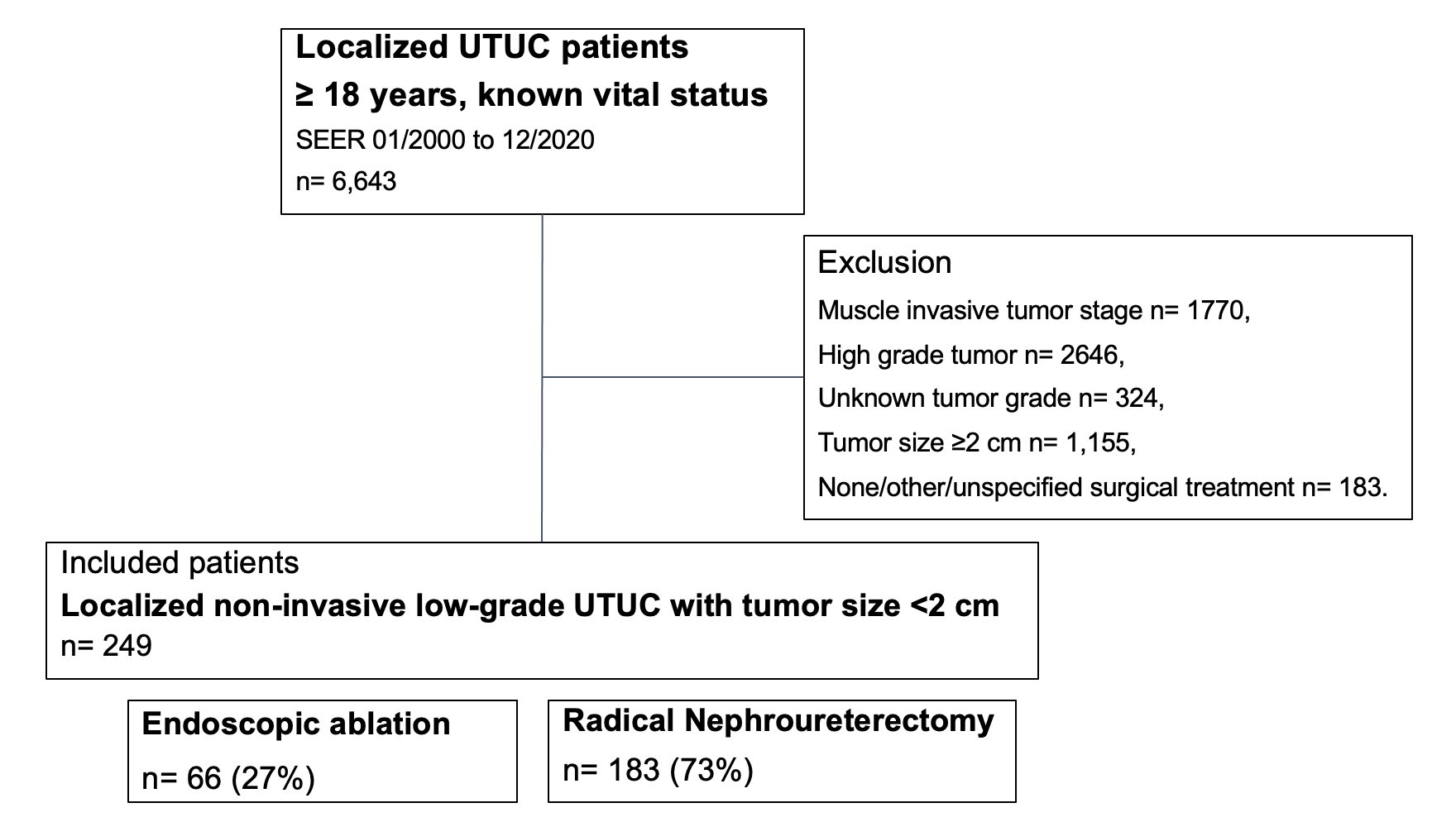

Supplement: Supplementary file 1 — Supplementary file1 (DOCX 177 KB) [file 345_2025_5626_MOESM1_ESM.docx]
